# Safety Assessment of Glucagon‐Like Peptide 1 Receptor Agonists Based on the FAERS Database: Focus on Tumorigenic Risk in Subpopulations

**DOI:** 10.1155/jdr/8893769

**Published:** 2026-03-20

**Authors:** Zhenhan Li, Jun Huang, Sha Zhou, Yongan Luan, Huang Xie, Zhongpei Chen, Juan Xu, Jun Qian

**Affiliations:** ^1^ Department of Endocrinology, Chongqing Traditional Chinese Medicine Hospital, Chongqing, China; ^2^ Chongqing Key Laboratory of Biochemistry and Molecular Pharmacology, Chongqing Medical University, Chongqing, China, cqmu.edu.cn; ^3^ Department of Cardiology, Tongji Hospital Affiliated to Tongji University School of Medicine, Shanghai, China, tongji.edu.cn

**Keywords:** adverse drug events, FAERS, glucagon-like peptide 1 receptor agonists, real-world data analysis, tumor-related risks

## Abstract

GLP‐1 receptor agonists (GLP‐1RAs) are extensively used in treating Type 2 diabetes due to their therapeutic benefits. However, clinical reports have highlighted adverse events, particularly the underexplored association with tumor development. This study investigates GLP‐1RA–related adverse events, focusing on tumorigenic risks, using data from the FDA Adverse Event Reporting System (FAERS). FAERS data from Q1 2005 to Q4 2023 were analyzed using the proportional reporting ratio (PRR) method to identify GLP‐1RA–associated adverse events. Detailed analyses examined event distribution across genders, ages, and body weights, with an emphasis on tumor‐related risks in vulnerable subpopulations. A total of 195,250 GLP‐1RA–related adverse event reports were identified, spanning 25 system organ classes (SOCs). The most reported SOCs were gastrointestinal disorders (*n* = 110,451, PRR 2.44), investigative findings (*n* = 80,126, PRR 2.46), and metabolism and nutrition disorders (*n* = 25,236, PRR 2.21). Notably, 7457 cases involved benign, malignant, and unspecified tumors (PRR 0.5). Further analysis revealed that the risk of GLP‐1RA–associated tumorigenesis varied by age, sex, and weight, suggesting that tumorigenesis risk differed in specific subpopulations. These findings underscore the need for vigilance regarding GLP‐1RA–related adverse events, particularly tumor risks in specific subgroups. Clinicians should consider patient‐specific factors, such as age, gender, and body weight, to ensure safe and effective GLP‐1RAs therapy.

**Summary**

• **What Is the Current Knowledge on the Topic?**GLP‐1 receptor agonists (GLP‐1RAs) are widely used for the treatment of Type 2 diabetes due to their significant therapeutic benefits. Although GLP‐1RAs have known adverse effects, the association between their use and tumor development remains underexplored. Understanding the demographic and physiological factors contributing to GLP‐1RA‐related adverse events is critical for optimizing their safe use.
• **What Question Did this Study Address?**What are the specific adverse events, particularly tumorigenic risks, associated with GLP‐1RA use, and how do these risks vary across different patient subpopulations based on gender, age, and body weight?

## 1. Introduction

According to the International Diabetes Federation (IDF), approximately 1 in 11 adults worldwide has diabetes, with Type 2 diabetes mellitus (T2DM) accounting for 90% of cases. As the number of people with diabetes is projected to reach 642 million by 2040, the prevalence of diabetes and its complications has become a major global health threat [1]. The primary drivers of the T2DM epidemic are multifactorial and include unhealthy dietary habits, sedentary lifestyles, population aging, economic development, and urbanization [2, 3]. Type 1 diabetes mellitus is generally managed with insulin replacement therapy, whereas T2DM is treated with oral hypoglycemic agents or a combination of oral agents and insulin. Commonly used hypoglycemic agents for T2DM are classified by their mechanisms of action, including insulin secretagogues, biguanides, insulin sensitizers, alpha‐glucosidase inhibitors, incretin mimetics, amylin antagonists, and sodium–glucose cotransporter‐2 (SGLT2) inhibitors [4–6]. Despite the significant therapeutic benefits of these conventional treatments, general adverse effects such as hypoglycemia, gastrointestinal distress, allergic reactions, hepatic and renal impairment, and cardiovascular events can lead to poor patient compliance and suboptimal treatment outcomes [7–9].

GLP‐1 receptor agonists (GLP‐1RAs), also known as glucagon‐like peptide‐1 receptor agonists, represent a newer class of hypoglycemic agents, including liraglutide and exenatide, which are primarily used to manage blood glucose levels in adults with T2DM [10, 11]. GLP‐1RAs work by binding to the GLP‐1 receptor, thereby slowing gastric emptying, suppressing appetite, enhancing insulin secretion, and inhibiting glucagon release, ultimately promoting glycemic control and weight management [11, 12]. For example, semaglutide, a weekly GLP‐1RA formulation, has shown superior efficacy in blood glucose reduction and weight loss in the RELATE‐OW and SEPRA studies, compared with other GLP‐1RAs [13]. Current diabetes management guidelines recommend prioritizing GLP‐1RAs for T2DM patients at high risk for cardiovascular and other diseases. Additionally, liraglutide and semaglutide have been approved in multiple countries for weight management, making them promising options for weight loss [14, 15]. However, the use of GLP‐1RAs is also linked to the risk of various adverse events (AEs), underscoring the need for a comprehensive evaluation of their efficacy and safety.

The United States Food and Drug Administration (FDA) Adverse Event Reporting System (FAERS) is a spontaneous reporting system for adverse drug events (ADEs), including data on AEs and medication errors collected by the FDA. By analyzing FAERS, previously unrecognized safety signals can be identified for timely and continuous risk management during clinical practice. In this study, we performed data mining on GLP‐1RA–related reports in the FAERS database to understand differences in ADEs among diverse real‐world populations. This study is further aimed at detecting signals of unknown, rare, and serious ADEs associated with GLP‐1RAs, providing a reference for GLP‐1RA selection in different populations and informing the management of their long‐term use.

## 2. Methods

### 2.1. Data Sources

Data for this study were obtained from the FAERS database, which is updated quarterly and includes patient demographics and management information (DEMO), drug utilization (DRUG), adverse reactions (REAC), patient outcomes (OUTC), reporting source information (RPSR), drug therapy start and end dates (THER), and drug indications (INDI). Considering the market entry of GLP‐1RAs, data were collected from the first quarter of 2005 to the third quarter of 2023 in the United States. FAERS data were collected and preprocessed using R software (Version 4.1.3), including coding and categorizing preferred terms (PTs) based on the Medical Dictionary for Regulatory Activities (MedDRA) AEs and system organ classes (SOCs) codes to analyze the SOC involved in AEs [16]. This study focused on examining ADEs where GLP‐1RAs were the primary suspect drugs, with additional analyses incorporating gender, age, and reporting source to evaluate subpopulation‐specific risks.

### 2.2. Data Extraction and Identification

ADEs related to GLP‐1RAs were identified through precise searches using the term “glucagon‐like peptide‐1 receptor agonists” and specific trade names, including “exenatide, liraglutide, bellarutide, losanatide, exenatide microspheres, somatostatin, abirateride, and Dulcolax.” Duplicate entries were identified and removed to ensure data accuracy. The categorization of reported AEs utilized the standard coding of PT as defined in MedDRA (Version 26.0).

### 2.3. Statistical Analysis

**Figure 1:**
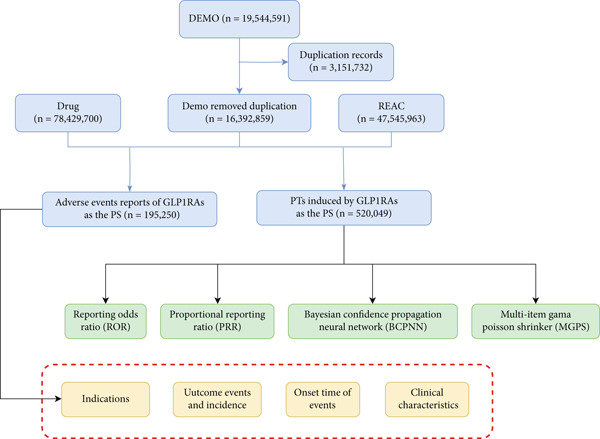
The flow diagram of selecting GLP1‐RAs–related AEs from FAERS database.

This study applied a comprehensive approach by using multiple algorithms, including ROR, PRR, BCPNN, and MGPS, to maximize the detection range, validate results from multiple perspectives, and identify more reliable safety signals. Specific criteria for determining positive signals using each algorithm refer to previous research [17]. Using this combined analytical approach, we detected FAERS signals from the first quarter of 2005 to the third quarter of 2023, with valid ADEs required to meet positive signal criteria across all mentioned algorithms. All data processing and statistical analyses were performed using R software (Version 4.1.3), and the analytical workflow is outlined in Figure 1.

## 3. Results

### 3.1. Descriptive Results for the Total Population

From the first quarter of 2005 to the third quarter of 2023, this study analyzed a total of 19,544,591 AEs from the FAERS database, of which 195,250 reports identified GLP‐1RAs as the primary suspect drug for ADEs. Among the AEs involving GLP‐1RAs, there was an overall increasing trend over time, with a higher proportion of female than male patients (57.69% vs. 36.97%). For age, a significant portion (45.82%) of reports lacked age data, limiting insights into age‐related trends. However, of the reports with specified age data, the age group of 60 years and above was the most common (30.05%). Most reports were submitted by consumers rather than healthcare professionals (76.25% vs. 16.43%), and the majority originated from the United States (68.75%). Regarding administration routes, subcutaneous injections were the most common (64.41%), followed by intravenous (2.46%), and oral routes (1.27%). Clinically, the most frequently reported serious ADEs were unspecified, but hospitalization or prolonged hospitalization was notably high (36.82%), followed by death (6.14%). Among cases that provided treatment duration, the highest percentages were for treatments of less than 7 days (23.63%) and greater than 60 days (10.31%). Specific formulas and thresholds are based on reference [17].

### 3.2. Basic Mining of Signal Detection Related to GLP‐1RAs

ADEs associated with GLP‐1RAs identified 25 SOCs related to these drugs. The most commonly affected systems were gastrointestinal disorders (*n* = 110,451, ROR 2.83, PRR 2.44, IC 1.27, EBGM 2.4), investigations (*n* = 80,126, ROR 2.73, PRR 2.46, IC 1.28, EBGM 2.43), and metabolism and nutrition disorders (*n* = 25,236, ROR 2.27, PRR 2.21, IC 1.13, EBGM 2.18), reflecting GLP‐1RAs′ endocrine pharmacology. Neoplasms, benign and malignant, were also reported (*n* = 7,457, ROR 0.5, PRR 0.5, IC −0.98, EBGM 0.51), warranting further attention as tumor‐related outcomes are not highlighted in the drug insert. Full details are in Table 1. We conducted a statistical analysis of the total number of adverse reactions associated with different types of GLP‐1RAs (see Figure S1). The results showed that exenatide had the highest number of adverse reactions, totaling 108,257 cases (48%), followed by liraglutide with 53,442 cases (23%), dulaglutide with 42,315 cases (19%), semaglutide with 12,160 cases (5%), and albiglutide with 11,278 cases (5%). Regarding the types of adverse reactions for individual drugs, we did not perform a detailed statistical analysis due to the fragmented and incomplete nature of the data for specific AEs associated with each medication.

**Table 1:** ADEs associated with GLP‐1RAs identified 25 SOCs.

**SOC**	**Case reports**	**ROR (95% CI)**	**PRR (95% CI)**	**Chi-square**	**IC (IC025)**	**EBGM (EBGM05)**
Gastrointestinal disorders	110,451	2.83 (2.81, 2.85)	2.44 (2.44, 2.44)	100,487.93	1.27 (1.26)	2.4 (2.39)
Investigations	80,126	2.73 (2.71, 2.75)	2.46 (2.46, 2.46)	72,488.35	1.28 (1.27)	2.43 (2.41)
Metabolism and nutrition disorders	25,236	2.27 (2.25, 2.3)	2.21 (2.17, 2.25)	16,743.81	1.13 (1.11)	2.18 (2.16)
Injury, poisoning, and procedural complications	64,605	1.4 (1.39, 1.41)	1.35 (1.35, 1.35)	6356.3	0.43 (0.42)	1.34 (1.34)
General disorders and administration site conditions	108,764	1.21 (1.2, 1.22)	1.17 (1.17, 1.17)	3112.11	0.22 (0.21)	1.16 (1.16)
Endocrine disorders	1186	0.89 (0.84, 0.94)	0.89 (0.84, 0.94)	16.56	−0.17 (−0.25)	0.89 (0.85)
Hepatobiliary disorders	3627	0.75 (0.72, 0.77)	0.75 (0.72, 0.78)	308.1	−0.41 (−0.46)	0.75 (0.73)
Eye disorders	7985	0.74 (0.72, 0.76)	0.74 (0.73, 0.75)	713.26	−0.42 (−0.45)	0.75 (0.73)
Ear and labyrinth disorders	1665	0.72 (0.68, 0.75)	0.72 (0.69, 0.75)	184.64	−0.47 (−0.54)	0.72 (0.69)
Nervous system disorders	30421	0.64 (0.63, 0.65)	0.66 (0.65, 0.67)	5732.39	−0.59 (−0.61)	0.66 (0.66)
Renal and urinary disorders	5607	0.55 (0.54, 0.57)	0.56 (0.55, 0.57)	1999.96	−0.84 (−0.87)	0.56 (0.55)
Neoplasms benign, malignant, and unspecified (incl cysts and polyps)	7457	0.5 (0.49, 0.51)	0.5 (0.49, 0.51)	3702.71	−0.98 (−1.01)	0.51 (0.5)
Skin and subcutaneous tissue disorders	13,753	0.46 (0.45, 0.47)	0.48 (0.47, 0.49)	8375.8	−1.06 (−1.09)	0.48 (0.47)
Musculoskeletal and connective tissue disorders	11,520	0.39 (0.38, 0.39)	0.4 (0.39, 0.41)	10,890.28	−1.31 (−1.34)	0.4 (0.4)
Cardiac disorders	5509	0.37 (0.36, 0.38)	0.38 (0.37, 0.39)	5747.34	−1.39 (−1.43)	0.38 (0.37)
Infections and infestations	10,835	0.37 (0.36, 0.38)	0.38 (0.37, 0.39)	11,216.41	−1.37 (−1.4)	0.39 (0.38)
Immune system disorders	2206	0.37 (0.35, 0.38)	0.37 (0.36, 0.38)	2385.64	−1.42 (−1.49)	0.37 (0.36)
Surgical and medical procedures	2532	0.35 (0.34, 0.37)	0.36 (0.35, 0.37)	2981.95	−1.48 (−1.54)	0.36 (0.35)
Psychiatric disorders	11,234	0.35 (0.34, 0.35)	0.36 (0.35, 0.37)	13,404.99	−1.46 (−1.48)	0.36 (0.36)
Vascular disorders	3916	0.33 (0.32, 0.34)	0.33 (0.32, 0.34)	5351.21	−1.58 (−1.62)	0.33 (0.33)
Respiratory, thoracic, and mediastinal disorders	8649	0.32 (0.32, 0.33)	0.34 (0.33, 0.35)	11,909.21	−1.56 (−1.59)	0.34 (0.33)
Reproductive system and breast disorders	1390	0.3 (0.29, 0.32)	0.31 (0.29, 0.33)	2193.27	−1.7 (−1.77)	0.31 (0.3)
Pregnancy, puerperium, and perinatal conditions	276	0.12 (0.1, 0.13)	0.12 (0.11, 0.13)	1841.34	−3.08 (−3.25)	0.12 (0.11)
Blood and lymphatic system disorders	960	0.1 (0.1, 0.11)	0.11 (0.1, 0.12)	7405.98	−3.23 (−3.32)	0.11 (0.1)
Congenital, familial, and genetic disorders	139	0.08 (0.07, 0.1)	0.08 (0.07, 0.09)	1429.25	−3.59 (−3.83)	0.08 (0.07)

At the PT level, four algorithms helped identify ADEs by ranking the Top 30 PTs based on the EBGM algorithm. High‐intensity PT signals included glycosylated hemoglobin abnormal (*n* = 220, ROR 36.12, PRR 36.1, IC 4.71, EBGM 26.09), insulin C‐peptide abnormal (*n* = 4, ROR 32.88, PRR 32.88, IC 4.61, EBGM 24.38), and glycosylated hemoglobin decreased (*n* = 278, ROR 27.28, PRR 27.27, IC 4.4, EBGM 21.18). Common side effects included blood glucose changes and injection site reactions, whereas thyroid‐ and pancreas‐related tumors appeared with higher frequency and signal intensity, suggesting further research may be warranted. Details are available in Table 2.

**Table 2:** ADEs by ranking the Top 30 PTs based on the EBGM algorithm.

**PT**	**Case reports**	**ROR (95% CI)**	**PRR (95% CI)**	**Chi-square**	**IC (IC025)**	**EBGM (EBGM05)**
Glycosylated hemoglobin abnormal	220	36.12 (30.89, 42.23)	36.1 (30.86, 42.23)	5366.54	4.71 (4.49)	26.09 (22.89)
Insulin C‐peptide abnormal	4	32.88 (10.47, 103.27)	32.88 (10.55, 102.48)	90.68	4.61 (3.16)	24.38 (9.36)
Glycosylated hemoglobin decreased	278	27.28 (23.85, 31.2)	27.27 (23.77, 31.28)	5404.51	4.4 (4.22)	21.18 (18.93)
Blood glucose	34	26.97 (18.39, 39.56)	26.97 (18.22, 39.91)	654.89	4.39 (3.86)	21 (15.24)
Blood glucose decreased	8694	24.61 (24.03, 25.2)	24.21 (23.74, 24.69)	152,748.87	4.27 (4.24)	19.31 (18.93)
Glycosylated hemoglobin increased	4221	24.41 (23.59, 25.25)	24.22 (23.29, 25.19)	74,133.6	4.27 (4.22)	19.31 (18.77)
Blood glucose increased	29,158	21.02 (20.75, 21.29)	19.9 (19.51, 20.29)	43,0367.4	4.04 (4.02)	16.49 (16.31)
Early satiety	720	77.89 (70.5, 86.06)	77.79 (70.53, 85.8)	29,338.96	5.4 (5.27)	42.28 (38.9)
Injection site nodule	3222	48.17 (46.15, 50.28)	47.88 (46.04, 49.79)	96,713.35	4.98 (4.93)	31.65 (30.54)
Injection site extravasation	3209	34.38 (33.01, 35.81)	34.17 (32.86, 35.54)	75,016.7	4.65 (4.59)	25.08 (24.24)
Sick building syndrome	3	30.14 (8.16, 111.34)	30.14 (8.11, 112.06)	63.39	4.51 (2.9)	22.86 (7.66)
Injection site indentation	244	22.85 (19.86, 26.3)	22.84 (19.91, 26.2)	4068.29	4.2 (4.01)	18.44 (16.39)
Injection site coldness	87	21.86 (17.29, 27.62)	21.85 (17.27, 27.64)	1394.26	4.15 (3.82)	17.79 (14.63)
Injection site injury	645	21.44 (19.67, 23.36)	21.41 (19.8, 23.16)	10,148.4	4.13 (4.01)	17.5 (16.29)
Intentional device misuse	1301	41.31 (38.68, 44.11)	41.21 (38.86, 43.71)	35,064.77	4.84 (4.75)	28.62 (27.09)
Accidental underdose	1990	36 (34.18, 37.92)	35.87 (33.82, 38.04)	48,302.6	4.7 (4.63)	25.97 (24.86)
Incorrect dose administered by device	2969	22.53 (21.64, 23.46)	22.41 (21.55, 23.31)	48,680.54	4.18 (4.13)	18.16 (17.56)
Drug dose titration not performed	252	22.35 (19.47, 25.66)	22.34 (19.48, 25.63)	4119.48	4.18 (3.98)	18.11 (16.14)
Post procedural hypothyroidism	32	21.12 (14.38, 31.04)	21.12 (14.27, 31.26)	497.25	4.11 (3.58)	17.31 (12.55)
Medullary thyroid cancer	99	66.82 (51.53, 86.64)	66.81 (51.78, 86.2)	3690.71	5.28 (4.94)	38.85 (31.26)
Huerthle cell carcinoma	8	23.34 (10.73, 50.77)	23.34 (10.66, 51.12)	135.95	4.23 (3.19)	18.75 (9.79)
Acinar cell carcinoma of pancreas	4	22.61 (7.56, 67.62)	22.61 (7.54, 67.76)	66.08	4.19 (2.79)	18.29 (7.31)
Adenocarcinoma pancreas	222	21.76 (18.79, 25.19)	21.75 (18.96, 24.95)	3542.58	4.15 (3.94)	17.73 (15.68)
Weight loss poor	652	29.21 (26.74, 31.9)	29.17 (26.45, 32.17)	13,412.73	4.48 (4.36)	22.3 (20.71)
Lack of satiety	33	25.73 (17.48, 37.87)	25.72 (17.38, 38.06)	610.52	4.34 (3.8)	20.25 (14.65)
Somogyi phenomenon	3	24.66 (6.88, 88.4)	24.66 (6.9, 88.16)	53.51	4.29 (2.7)	19.59 (6.73)
Eosinophilic panniculitis	3	38.75 (10.02, 149.87)	38.75 (10.02, 149.83)	77.24	4.78 (3.13)	27.43 (8.84)
Needle track marks	63	25.32 (19.15, 33.48)	25.32 (19.24, 33.31)	1149.7	4.32 (3.93)	20 (15.83)
Eructation	3216	28.25 (27.15, 29.4)	28.08 (27, 29.2)	64,112.82	4.44 (4.38)	21.67 (20.96)
Thyroid C‐cell hyperplasia	4	72.34 (19.43, 269.4)	72.34 (19.46, 268.96)	156.34	5.34 (3.8)	40.63 (13.52)

### 3.3. In‐Depth Mining of ADE Signal Detection by Gender, Age, and Body Weight

Exploring ADE signals across different demographic strata revealed insights into signal variation by gender, age, and body weight. First, in the analysis of gender differences, certain PT signals were found to be more prevalent among women, such as “blood glucose decreased,” “decreased appetite,” “diarrhea,” “nausea,” and “vomiting.” Conversely, PT signals such as “blood glucose increased,” “incorrect dose administered,” “injection site reactions,” “hemorrhage,” and “weight decreased” appeared more frequently in men.

In age‐stratified analysis, the Top 10 PT signals were examined across different age groups. In the 18–30 age group, PT frequencies, from highest to lowest, included “nausea,” “vomiting,” “diarrhea,” “blood glucose increased,” “weight decreased,” “injection site pain,” “decreased appetite,” and “injection site hemorrhage.” As age increased, “nausea,” “vomiting,” and “diarrhea” showed a decreasing trend, whereas the other PTs increased, with “blood glucose increased” and “weight decreased” showing the most significant increase with age. Thus, ADE signals varied significantly by age.

When analyzing ADE signals by body weight, the group weighing less than 50 kg displayed PT frequencies ranked as follows: “weight decreased,” “nausea,” “blood glucose increased,” “vomiting,” “decreased appetite,” “injection site pain,” “injection site hemorrhage,” and “diarrhea”. Notably, “weight decreased,” “nausea,” and “blood glucose increased” consistently showed higher frequencies across weight groups, whereas “incorrect dose administered” showed the lowest frequency across all body weights. This analysis highlights that while there are differences in PT signals across body weights, these variations were not substantial. All details are presented in Figure 2.

Figure 2In‐depth mining of ADE signal detection by gender, age, and body weight. (a) Adverse drug reactions in GLP‐1RA–related gender stratification. (b) Adverse drug reactions in GLP‐1RA–related age stratification. (c) Adverse drug reactions in GLP‐1RA–related body weight stratification.

**Figure:**
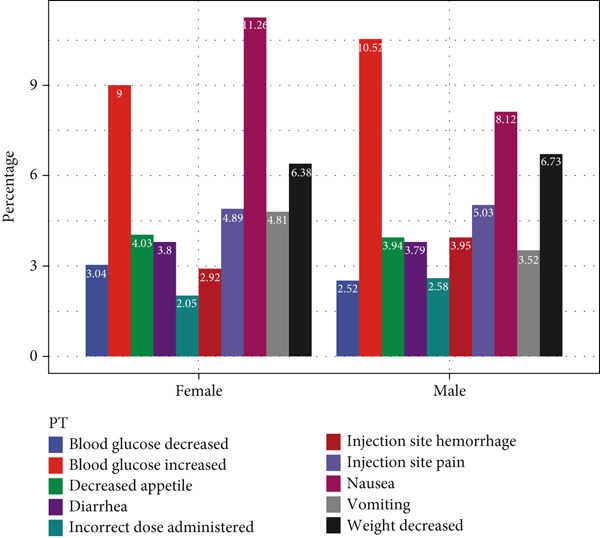
(a)

**Figure:**
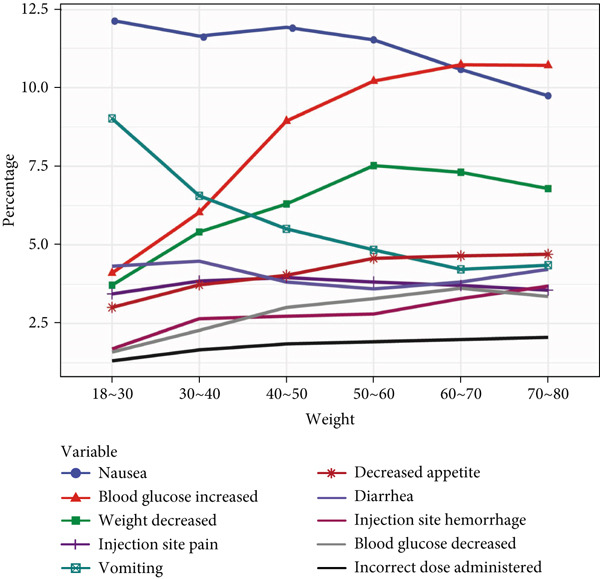
(b)

**Figure:**
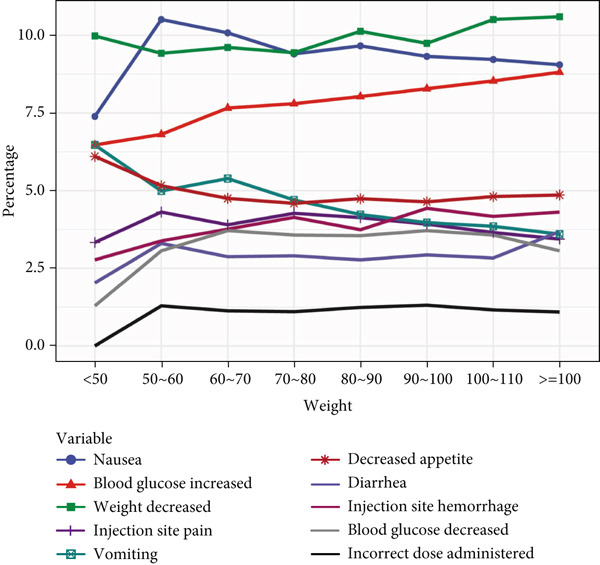
(c)

### 3.4. In‐Depth Analysis of GLP‐1RA‐Related Metabolism and Nutrition Disorders

Further analysis of GLP‐1RA–related ADEs showed that metabolism and nutrition disorders were prominent. We explored PT signals for these disorders across gender, age, and weight strata. Signals such as “feeding disorder,” “hypophagia,” “increased appetite,” and “weight loss poor” were more frequent in women. In contrast, “decreased appetite,” “dehydration,” “diabetes mellitus inadequate control,” “diabetic ketoacidosis,” “hyperglycemia,” and “hypoglycemia” were more common in men, suggesting gender‐specific signal patterns.

Age analysis of PT signals related to metabolism and nutrition showed that “decreased appetite” was prevalent across age groups, increasing notably with age, whereas other signals like “dehydration,” “diabetic ketoacidosis,” and “hypoglycemia” varied less significantly by age. In body weight analysis, “decreased appetite” remained the most frequent signal, showing a positive trend with increasing weight, whereas other PTs showed minimal variation across weights. All details are presented in Figure 3.

Figure 3In‐depth analysis of GLP‐1RA–related metabolism and nutrition disorders. (a) A deep‐potential analysis of metabolic‐related adverse drug reactions based on gender stratification. (b) A deep‐potential analysis of metabolic‐related adverse drug reactions based on age stratification. (c) A deep‐potential analysis of metabolic‐related adverse drug reactions based on body weight stratification.

**Figure:**
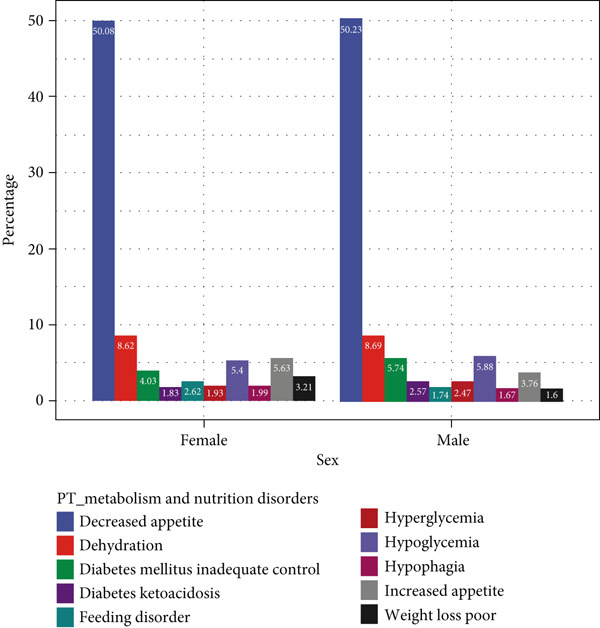
(a)

**Figure:**
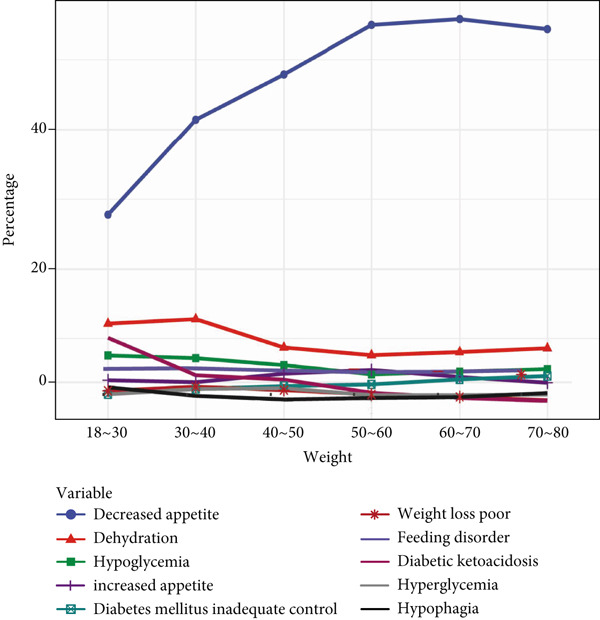
(b)

**Figure:**
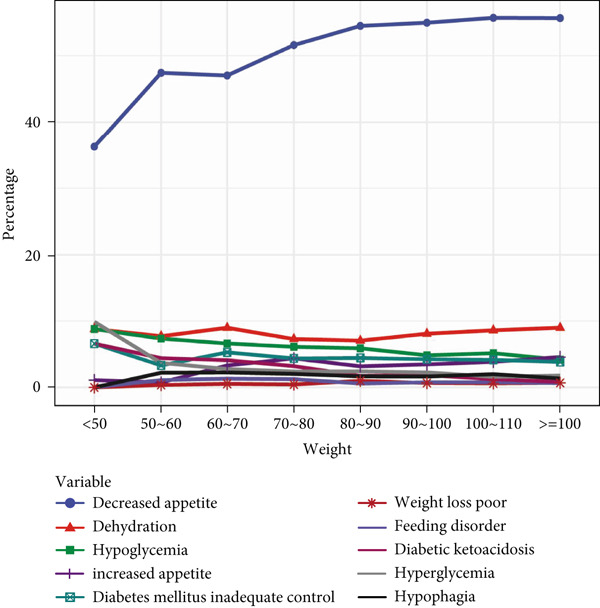
(c)

### 3.5. In‐Depth Analysis of GLP‐1RA‐Related Gastrointestinal Disorders

Gastrointestinal disorders represented a major category of ADEs associated with GLP‐1RAs. The PT signals “abdominal pain upper,” “nausea,” and “vomiting” were more common in women, whereas “abdominal discomfort,” “abdominal distension,” “abdominal pain,” “constipation,” “diarrhea,” “dyspepsia,” and “pancreatitis” were more prevalent among men. Age‐stratified analysis revealed that “nausea,” “vomiting,” and “diarrhea” were consistently reported across ages, with “nausea” and “diarrhea” increasing slightly with age, whereas “vomiting” decreased. Body weight analysis indicated similar trends for “nausea,” “vomiting,” and “diarrhea” across weight groups. Figure 4 details these findings.

Figure 4In‐depth analysis of GLP‐1RA–related gastrointestinal disorders. (a) A deep‐potential analysis of gastrointestinal‐related adverse drug reactions based on gender stratification. (b) A deep‐potential analysis of gastrointestinal‐related adverse drug reactions based on age stratification. (c) A deep‐potential analysis of gastrointestinal‐related adverse drug reactions based on body weight stratification.

**Figure:**
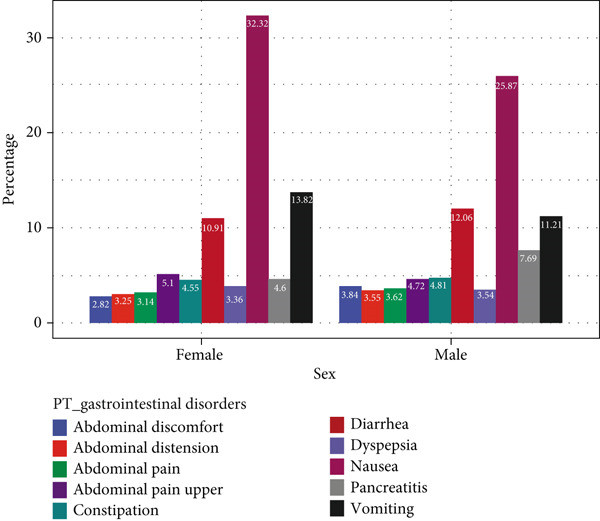
(a)

**Figure:**
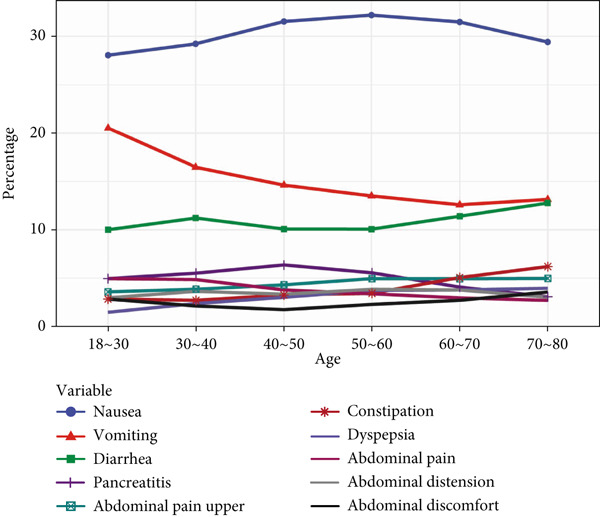
(b)

**Figure:**
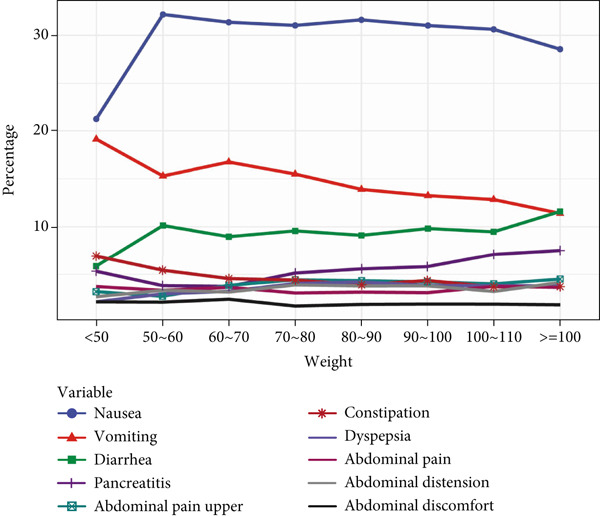
(c)

### 3.6. Analysis of Tumor‐Related ADEs With GLP‐1RAs

Tumor‐related AEs associated with GLP‐1RAs, although not prominent in the drug′s insert, showed an upward trend over time. Gender‐specific analysis showed that “neoplasm malignant,” “papillary thyroid cancer,” “thyroid cancer,” and “thyroid neoplasm” were more common in females, with “breast cancer” only observed in female patients, whereas “adenocarcinoma pancreas,” “metastases to liver,” “pancreatic carcinoma,” and “pancreatic carcinoma metastatic” were more frequent in men, with “prostate cancer” exclusive to male patients. Age‐stratified analysis indicated that thyroid‐related cancers were more frequent in younger age groups, whereas pancreatic tumors became more common with advancing age. Body weight analysis suggested that “pancreatic carcinoma” and “pancreatic carcinoma metastatic” were the most frequent PT signals, with other tumor types showing minimal weight‐related differences. All details are presented in Figure 5. Upon examining the temporal trends in reporting, we observed a mild decline in the volume of GLP‐1RA–related reports during 2021–2023, which may reflect the widespread disruptions in healthcare systems and AE reporting during the initial phase of the COVID‐19 pandemic. However, reporting volumes remained stable between 2020 and 2021. Importantly, the overall distribution of SOCs and the signal strength of key PTs remained consistent during this period, suggesting that the pandemic likely had minimal impact on the core safety signals identified in this study. A temporal analysis of the two most notable tumor types, pancreatic cancer and thyroid cancer, revealed a shifting trend around the Year 2015. The number of reported cases for both malignancies demonstrated a gradual increase up until 2015, followed by a marked decrease in the subsequent years (Figure S2).

Figure 5Analysis of tumor‐related ADEs with GLP‐1RAs. (a) A deep‐potential analysis of tumor‐related adverse drug reactions based on time. (b) A deep‐potential analysis of tumor‐related adverse drug reactions based on gender stratification. (c) A deep‐potential analysis of tumor‐related adverse drug reactions based on age stratification. (d) A deep‐potential analysis of tumor‐related adverse drug reactions based on body weight stratification.

**Figure:**
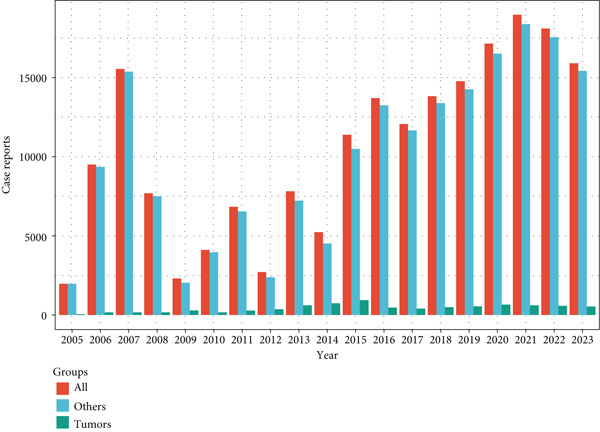
(a)

**Figure:**
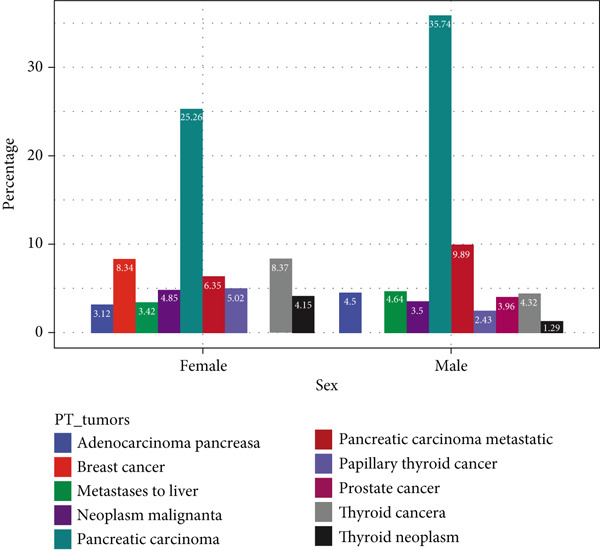
(b)

**Figure:**
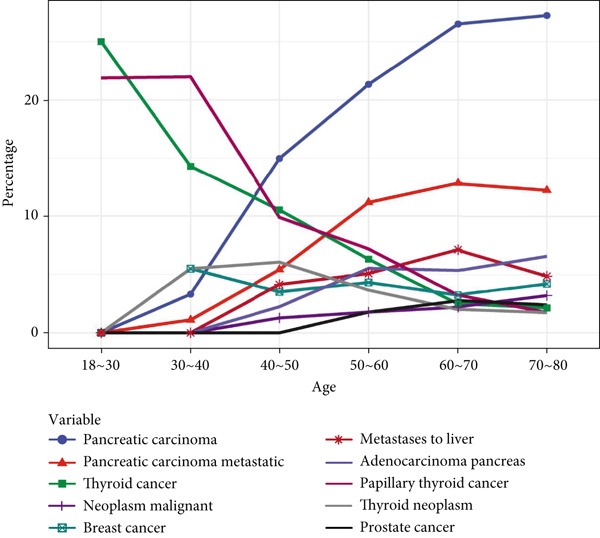
(c)

**Figure:**
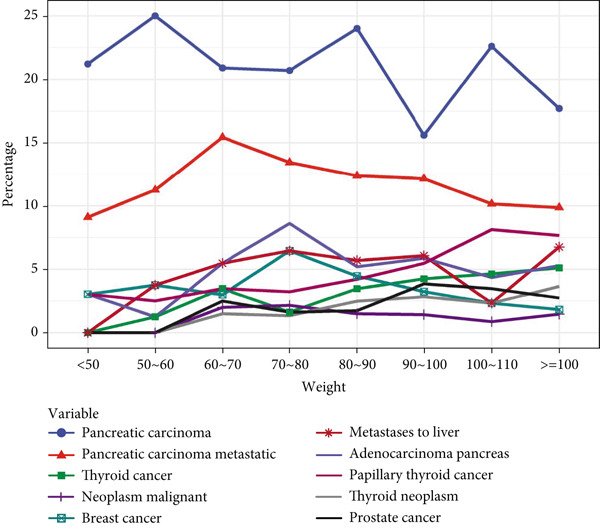
(d)

## 4. Discussion

GLP‐1RAs are a class of drugs widely recommended for treating T2DM. These agents exert glucose‐lowering effects by agonizing the glucagon‐like peptide‐1 receptor, which has an enteroglucagon action while also acting as insulinotropic agents that promote weight reduction [10]. Additionally, GLP‐1RAs have been shown to lower the risk of MACE, all‐cause mortality, hospitalization for heart failure, and worsening renal function in T2DM patients [18, 19]. Due to these benefits, GLP‐1RAs are broadly endorsed for managing T2DM [20–23]. However, as noted in the drug′s instructions, GLP‐1RAs carry potential adverse effects, particularly gastrointestinal issues like nausea, vomiting, and diarrhea, as well as thyroid‐ and pancreas‐related tumors. This study utilizes the FAERS database to thoroughly assess GLP‐1RA‐associated AEs and examine their safety profile.

Of the 25 SOC of drug‐related adverse reactions of GLP‐1RAs found in our study, the study showed that the three most common systems were gastrointestinal disorders, investigations, and metabolism and nutrition disorders, which is consistent with the characterization of GLP‐1RAs as endocrinology drugs. Among them, gastrointestinal disorders had the highest incidence of adverse reactions. We then performed an in‐depth analysis of overall, gastrointestinal disorders, and metabolism and nutrition disorders, and found that PTs differed by age, gender, and body weight. Of interest, benign, malignant, and unspecified tumors were also important AEs present in GLP‐1RAs, although tumor‐related disorders were not emphasized in the drug insert. Studies have found that the incidence of tumor‐related diseases has largely been on the rise in recent years. Tumor malignancy, papillary thyroid cancer, thyroid cancer, and thyroid tumors are more common in women, while breast cancer occurs exclusively in female patients. Adenocarcinoma of the pancreas, liver metastases, pancreatic cancer, and pancreatic metastases are more common in men. Thyroid cancer and papillary thyroid cancer had the highest incidence in the age group of 18–30 years, but both tended to decrease with age, and pancreatic cancer and pancreatic cancer metastasis increased significantly with age, becoming the two most common PTs from the age of 50 years onwards. The two most common PTs were pancreatic cancer and pancreatic cancer metastasis at different body weights.

A systematic evaluation and network meta‐analysis of GLP‐1RAs in patients with T2DM found that although GLP‐1RAs were effective in terms of glycemia and body weight, they induced gastrointestinal AEs, especially when administered at high doses [24]. Similarly, several other studies on GLP‐1RA have mentioned the risk of GLP‐1RA–associated gastrointestinal AEs in addition to affirming the efficacy of the drug [25–27]. This is consistent with our current findings that gastrointestinal AEs occupy the top spot of GLP‐1RA ADEs. In addition, due to the widespread use of GLP‐1RA, safety concerns have arisen, especially in tumor AEs. In a nested case‐control analysis of patients with T2DM who were treated with second‐line anti‐diabetic drugs between 2006 and 2018 in the French National Health Insurance System (SNDS) database, Bezin et al. found that the use of GLP‐1 RA increases the risk of all thyroid and medullary thyroid cancers, especially 1–3 years after treatment [28]. Several other clinical studies have also confirmed that GLP‐1 RA increases the risk of colorectal cancer and pancreatic tumors in patients with Type 2 diabetes [29, 30]. Yang et al. conducted an analysis of GLP‐1 agonist‐related tumor AEs and found that GLP‐1RA was associated with benign and malignant thyroid tumors and pancreatic malignancies at the HLT level, whereas bile duct cancer and breast cancer were controversial [31]. Therefore, GLP‐1RAs may increase the risk of certain tumors, and the risk of different tumors varies according to age, sex, and weight. Our study provides an in‐depth analysis of this, and it is important to focus on different tumorigenesis when treating different subpopulations of GLP‐1RAs. In contrast to the potential tumorigenic risks identified in our analysis, a growing body of evidence suggests that GLP‐1RAs may conversely confer a protective effect against certain obesity‐associated cancers. This seemingly paradoxical effect is biologically plausible, given the well‐established link between obesity and the pathogenesis of several malignancies. For instance, a recent large‐scale cohort study by Dai et al. [32] indicated that the use of GLP‐1RAs in adults with obesity was associated with a significantly reduced risk of specific cancers, including endometrial and ovarian cancers, compared with non‐GLP‐1RA anti‐obesity medications. This protective effect is likely mediated, at least in part, through substantial and sustained weight loss, improved metabolic parameters, and reduction in chronic inflammation. Therefore, the relationship between GLP‐1RAs and cancer risk appears to be complex and site‐specific. Altough our data and other studies signal a need for vigilance regarding thyroid and pancreatic tumors, the net oncological impact of these agents might be beneficial in a broader patient context, particularly for those with obesity. Future long‐term, prospective studies are warranted to clarify these dual and organ‐specific effects.

The findings of this study carry significant implications for the clinical management of patients treated with GLP‐1RAs. To mitigate potential risks and optimize therapeutic outcomes, a proactive and personalized approach is warranted. Firstly, baseline psychiatric screening should be considered prior to initiating therapy, particularly for patients with a known history of mental health disorders. Secondly, clinicians should advocate for ongoing monitoring of patients throughout treatment, maintaining vigilance for emergent mood changes, suicidal ideation, and other psychiatric symptoms. Thirdly, comprehensive patient education is crucial; patients should be informed about potential adverse effects, including psychiatric ones, and encouraged to report any concerning symptoms promptly. Finally, managing complex cases may require a multidisciplinary care model, involving collaboration among endocrinologists, psychiatrists, and primary care physicians to ensure both metabolic and mental health are adequately addressed. These strategies, centered on vigilance and personalized care, are essential for harnessing the benefits of GLP‐1RAs while safeguarding patient safety in diverse subpopulations.

## 5. Conclusion

GLP‐1RAs have shown considerable glucose‐lowering efficacy, minimal hypoglycemia risk, and notable cardiovascular protective effects, underscoring their clinical value and promising application potential. However, these drugs are also associated with risks, particularly tumor‐related risks that are noted but not heavily emphasized in usage guidelines. Using the FAERS database, we conducted a comprehensive evaluation of GLP‐1RAs safety, with a particular focus on tumor associations. Our analysis indicated varying tumor risks among different subpopulations exposed to GLP‐1RAs. Therefore, in clinical practice, it is crucial to assess both the benefits and risks of GLP‐1RAs on an individual basis, make informed therapeutic decisions, and implement personalized disease management strategies.

## 6. Limitations

Our study was limited by the nature of the FAERS database. As a spontaneous reporting system, it lacks comprehensive information on patient histories that may influence AE risks. This includes past medical history (e.g., psychiatric diagnoses), family history, personal lifestyle factors (e.g., smoking and alcohol consumption), and occupational exposures. Furthermore, data on concomitant medications are often incomplete or inconsistently reported. The absence of these critical confounders may impact the accuracy of our findings, particularly for outcomes like tumorigenesis and psychiatric events, and precludes reliable analysis of drug–drug interactions or the role of underlying conditions. As a spontaneous reporting system, FAERS is susceptible to external influences that can affect reporting rates. As noted in the results, we observed a transient decrease in reporting volume during the stages of the COVID‐19 pandemic. Although our analysis indicated that the primary safety signals remained consistent, we cannot entirely rule out the possibility that some nondifferential underreporting during this period may have influenced the absolute number of certain events, particularly those that are less severe or require in‐person diagnosis.

## Conflicts of Interest

The authors declare no conflicts of interest.

## Author Contributions

Zhenhan Li and Jun Huang wrote the manuscript. Zhenhan Li, Jun Qian, Juan Xu, and Sha Zhou designed the research. Zhenhan Li, Jun Qian, Yongan Luan, Huang Xie, Zhongpei Chen, and Juan Xu performed the research and analyzed the data. Jun Qian and Juan Xu contributed equally and shared the last authorship.

## Funding

This study was supported by the Key Project of Chongqing Municipal Technological Innovation and Application Development Special Fund (No. CSTB2025TIAD‐KPX0096); the Chongqing Medical Youth Top Talent Project (No. YXQN202441); the Chongqing Municipal Science and Health Joint Medical Research Project (No. 2023QNXM002); the 4th Young Talent Project of Chongqing Traditional Chinese Medicine Hospital; the Clinical Research Project of Tongji Hospital of Tongji University (No. ITJ(QN)2203); and the Chongqing Postdoctoral Innovative Talents Support Program Fund (No. CQBX202212).

## Supporting Information

Additional supporting information can be found online in the Supporting Information section.

## Supporting information


**Supporting Information 1** Figure S1: Adverse reactions associated with different types of GLP‐1RAs (Pie chart).

**Supporting Information 2** Figure S2: Temporal trends in GLP‐1RA–related adverse event reports and incidence of pancreatic and thyroid cancers.

## Data Availability

These data were derived from the following resources available in the public domain: (FDA Adverse Event Reporting System [FAERS] and https://www.fda.gov/drugs/fdas-adverse-event-reporting-system-faers/fda-adverse-event-reporting-system-faers-latest-quarterly-data-files).
