# Supplementary figures and images for: Safety Assessment of Glucagon‐Like Peptide 1 Receptor Agonists Based on the FAERS Database: Focus on Tumorigenic Risk in Subpopulations
Source: J Diabetes Res. 2026 Mar 20;2026:8893769. doi: 10.1155/jdr/8893769 (PMC13140801; doi:10.1155/jdr/8893769)

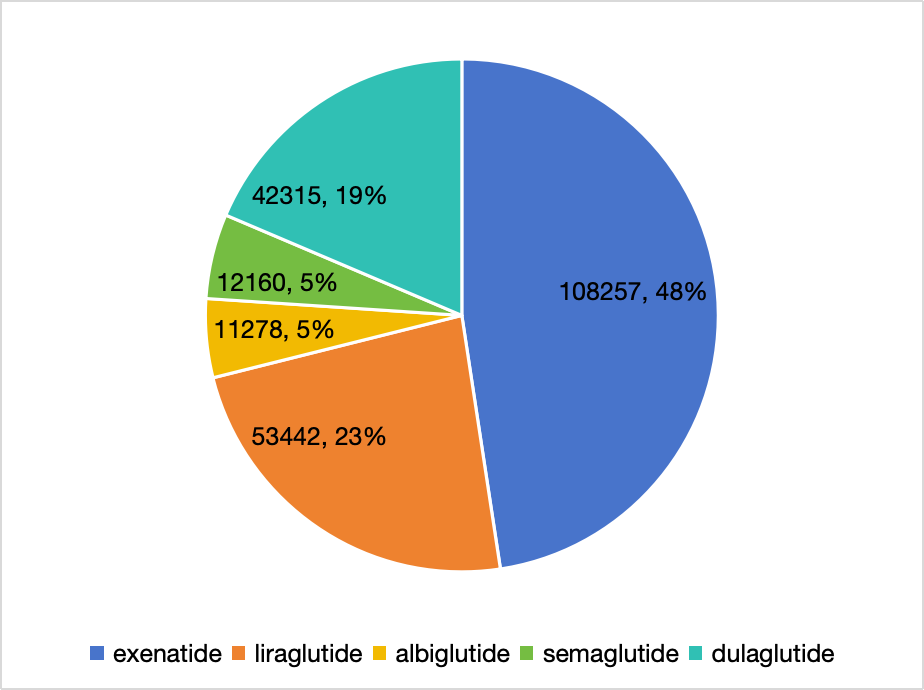

Supplement: Supplementary file 1 — Supporting Information 1 Figure S1: Adverse reactions associated with different types of GLP‐1RAs (Pie chart). [file JDR-2026-8893769-s001.png]

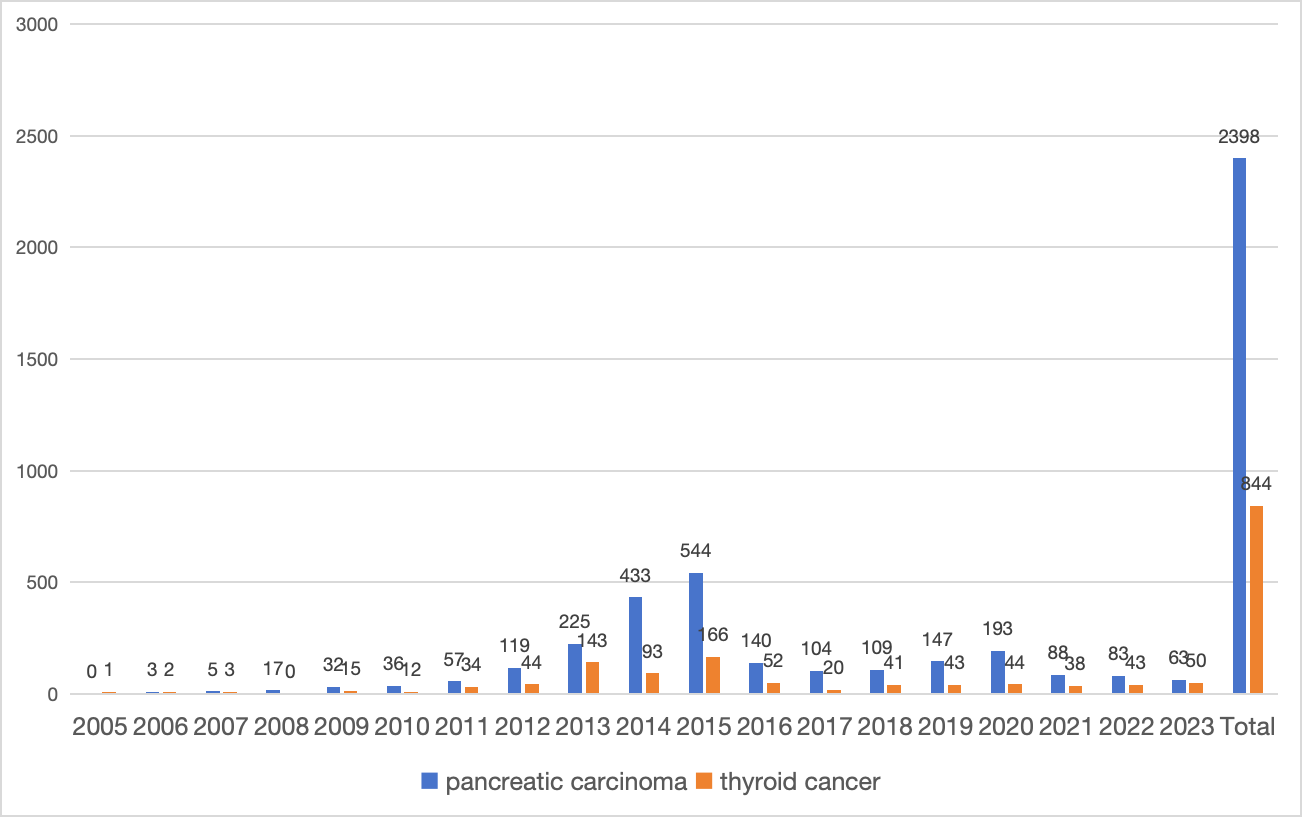

Supplement: Supplementary file 2 — Supporting Information 2 Figure S2: Temporal trends in GLP‐1RA–related adverse event reports and incidence of pancreatic and thyroid cancers. [file JDR-2026-8893769-s002.tif]
